# Fine Mapping of the Body Fat QTL on Human Chromosome 1q43

**DOI:** 10.1371/journal.pone.0153794

**Published:** 2016-04-25

**Authors:** Brahim Aissani, Howard W. Wiener, Kui Zhang

**Affiliations:** 1 Department of Epidemiology, University of Alabama at Birmingham School of Public Health, Birmingham, Alabama, United States of America; 2 Department of Mathematical Sciences, Michigan Technological University, Houghton, Michigan, United States of America; Wake Forest School of Medicine, UNITED STATES

## Abstract

**Introduction:**

Evidence for linkage and association of obesity-related quantitative traits to chromosome 1q43 has been reported in the Quebec Family Study (QFS) and in populations of Caribbean Hispanic ancestries yet no specific candidate locus has been replicated to date.

**Methods:**

Using a set of 1,902 single nucleotide polymorphisms (SNPs) genotyped in 525 African American (AA) and 391 European American (EA) women enrolled in the NIEHS uterine fibroid study (NIEHS-UFS), we generated a fine association map for the body mass index (BMI) across a 2.3 megabase-long interval delimited by *RGS7* (regulator of G-protein signaling 7) and *PLD5* (Phospholipase D, member 5). Multivariable-adjusted linear regression models were fitted to the data to evaluate the association in race-stratified analyses and meta-analysis.

**Results:**

The strongest associations were observed in a recessive genetic model and peaked in the 3’ end of *RGS7* at intronic rs261802 variant in the AA group (p = 1.0 x 10^−4^) and in meta-analysis of AA and EA samples (p = 9.0 x 10^−5^). In the EA group, moderate associations peaked at rs6429264 (p = 2.0 x 10^−3^) in the 2 Kb upstream sequence of *RGS7*. In the reference populations for the European ancestry in the 1,000 genomes project, rs6429264 occurs in strong linkage disequilibrium (D’ = 0.94) with rs1341467, the strongest candidate SNP for total body fat in QFS that failed genotyping in the present study. Additionally we report moderate associations at the 3’ end of *PLD5* in meta-analysis (3.2 x 10^−4^ ≤ p ≤ 5.8 x 10^−4^).

**Conclusion:**

We report replication data suggesting that *RGS7*, a gene abundantly expressed in the brain, might be a putative body fat QTL on human chromosome 1q43. Future genetic and functional studies are required to substantiate our observations and to potentially link them to the neurobehavioral phenotypes associated with the *RGS7* region.

## Introduction

The genetic basis of obesity in humans has been the focus of numerous and intense genome-wide linkage scans and association studies over the last two decades. Obesity is a common multifactorial condition resulting from an imbalance between energy intake and energy expenditure. Its prevalence increased over the last decades, reaching epidemic proportions in the U.S. adult population [1, 2]. Importantly, obesity is a major risk factor for chronic diseases such as type 2 diabetes, hypertension, and coronary heart disease [3]. Although environmental factors are major promoter of obesity, strong heritability of anthropometric measures of obesity such as the body mass index (BMI) (heritability h^2^ = 0.77) has been reported.

Early studies explored the genetic basis of obesity with the assumption that an oligogenic model of pathogenesis underlies the development of this highly prevalent condition in contemporary human populations. While an increasing number of genetic loci causing Mendelian forms of obesity have been identified—including but not limited to leptin (*LEP*), proopiomelanocortin (*POMC*), melanocortin 4 receptor (*MC4R*) in early studies, and single-minded homolog 1 (*SIM1*), brain-derived neurotrophic factor (*BDNF*) and its receptor encoded by the neurotrophic tyrosine kinase receptor type 2 gene (*NTRK2*) in later studies [4–6]; these genetic loci are associated with morbid forms of obesity and explain only a small proportion of the observed obesity in populations.

In a previous linkage scan and follow-up linkage disequilibrium (LD) mapping in the Quebec Family Study (QFS), we mapped a quantitative trait locus (QTL) influencing total body adiposity to the 5’UTR of *RGS7* on chromosome 1q43 [7]. *RGS7* is not listed among the candidate loci identified in genome-wide linkage or association studies of obesity-related phenotypes in humans; however, loci influencing these phenotypes or metabolic-related markers map close to *RGS7*. For instance, A genome-wide linkage scan in Caribbean Hispanics identified chromosome 1q43 at D1S547 (about 233 Kb telomeric to *RGS7*) as the strongest signal for body weight and as the second strongest signal for BMI [8]. A GWAS of childhood obesity in Hispanic populations showed nominal association of *RGS7* with fasting plasma vitamin B12 [9], a marker of obesity [10]. A more recent GWAS in Europeans identified *SDCCAG8* (serologically defined colon cancer antigen 8) on 1q43 as a novel gene for early-onset extreme obesity (body weight phenotype) [11].

In the present study, we re-assessed the association of BMI with a dense set of chromosome 1q43 SNPs in a sample of 916 women enrolled in the NIEHS uterine fibroid study and report peak of associations in *RGS7* in race-stratified analyses and in meta-analysis.

## Material and Methods

### Ethics Statement

The NIEHS-UFS and the present ancillary study were approved by the Human Subjects Review Boards at the NIEHS, George Washington University and University of Alabama at Birmingham, respectively. Participants gave written informed consent in accordance with these Review Boards.

### Study population

Detailed characteristics of the study population have been reported [12, 13]. Briefly, a random sample of women, aged 35 to 51 years, was selected from a computerized list of members of a prepaid urban health plan for enrollment in the NIEHS-UFS [12]. Of the enrolled premenopausal women, 1,045 (93%) had available DNA specimens and self-reported African American (AA, n = 574), non-Hispanic European American (EA, n = 394) or other (n = 77) ethnic background.

### Study outcome and covariates

Normalized body mass index (BMI), defined as weight in kilograms divided by height in meters squared (Kg/m^2^) was used as a surrogate measure of obesity. Covariates were age and physical activity, and because the primary study outcome in NIEHS-UFS is uterine fibroids, the fibroid affection status was also included as a covariate.

Data on physical activity was collected through computer-assisted telephone interview [14]. Participants were asked for the time (numbers of hours) spent per week in each of three categories of physical activity unrelated to occupation: 1) vigorous activity such as running, 2) moderate activity such as dancing and 3) walking. Less detailed data were collected about non-occupational physical activity at age 30 years and as a teenager. The data for each time period were combined into summary variables based on estimated metabolic rates for each category of physical activity [15] and were then standardized to vigorous activity [14].

### Genotyping

#### DNA preparation

DNA was extracted from blood samples using the QIAAMP DNA Mini Kit (Qiagen, Valencia, California) procedure and isolated DNA has undergone purification prior to quantification by the PicoGreen^®^ assay (Invitrogen™).

Genotyping. Genotype data were available for a combined set of 1,902 quality control-filtered SNPs typed previously in a two-stage genetic association study [13, 16]. Illumina® iSelect and Illumina®GoldenGate platforms (Illumina Inc., San Diego, CA) were used for SNP genotyping in the two-stage study. Reliability in genotyping data was assessed by inclusion of blind duplicates (2 duplicates per 96-well plates) and HapMap positive control samples (4 controls per 96-well plate) as required by the Genomic Core at the HudsonAlpha Institute (Hunstville, AL) and Johns Hopkins University (Maryland, MD).

### Statistical analysis

#### Quality control

A call rate of 95% and a concordance rate > 99.0% between duplicates were assigned as quality control thresholds of the genotyping data. Prior to their inclusion in the analysis, SNP calls were examined separately in each ethnic group for adherence to Hardy-Weinberg equilibrium (HWE) using the Pearson’s chi-squared test and SNPs showing significant deviation (p < 0.01) from HWE were excluded.

#### Association testing

Model-free Discriminant Analysis of Principal Components (DAPC) [17] based on a total set of 4,363 SNPs from over the genome was used in a previous study to define clusters of genetically related individuals in NIEHS-UFS [13]. General linear models (GLM) adjusted for covariates were fitted to the data to evaluate the association between SNP genotypes and body mass index modeled as a continuous outcome. Physical activity was categorized as low (lowest 33 percent of the combined African-American and White distribution), medium (middle 33 percent), high (67^th^-83^rd^ percentile) and very high (above the 83^rd^ percentile) [14]. SNP associations were tested under additive, dominant and recessive genetic models.

P-values from two-sided F-tests were provided to assess the statistical significance of each SNP in race-stratified analyses. Meta-analysis was conducted using random-effect DerSimonian-Laird estimator [18] in the Metafor package [19] from R (https://www.r-project.org/).

We report only the SNPs that reached significance levels below the threshold p < 0.01 in any of the tested models for the predominant EA and AA groups or in meta-analysis of these groups. Given the correlation among the dense set of SNPs used in the fine mapping study, correction for multiple testing was based on the effective number of SNPs, which was estimated for each study population using SimpleM [20]. P-values below the corrected threshold (0.05/787 = 6.3 x 10^−5^ in EA and 0.05/1,233 = 4.1 x 10^−5^ in AA) were deemed to be statistically significant.

## Results

Except for a unique individual of European descent, typing was achieved in the entire study population (n = 1,045). Among the typed samples, about 92% have call rates greater than 98%. Forty-two individuals (4.0%) with call rates < 95% were excluded from the analysis; this yielded a total of 1,003 samples for analysis. The overall concordance rate between duplicates was 99.6%, implying that false discovery due to typing errors is unlikely. Discriminant analysis re-assigned several individuals who self-identified as “other populations” to either EA or AA ancestry (**S1 Fig**). Sixteen individuals who self-identified as African American (AA) and one individual as a non-Hispanic European American (EA) clustered more closely with the Yoruban African population (YRI); these 17 individuals were excluded from further analyses. Further exclusion of 70 individuals who self-reported race/ethnicity as “other” or “mixed origins” resulted in a total of 916 individuals (525 AA and 391 EA) available for analysis.

**Fig 1:**
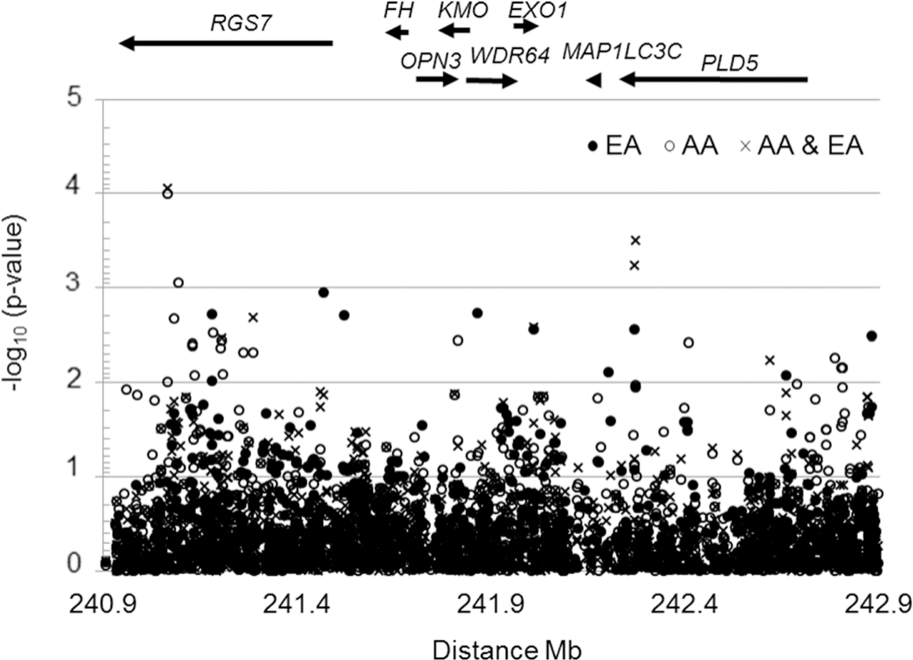
Fine genetic association map of body mass index on chromosome 1q43 in NIEHS uterine fibroid study. The plot shows the significance level of SNP associations (expressed as minus log_10_ of p-value) with the body mass index (BMI) in a sample of 525 African American (EA) and 391 European American (EA) women participants in NIEHS-UFS (National Institute of Environmental Health Science-Uterine Fibroid Study). P-values were obtained from general linear models adjusted for age, physical activity and uterine fibroid affection status. The results were obtained under the assumption of a recessive genetic model in race-stratified analyses and in meta-analysis using beta coefficient and standard error estimates from the latter models. *RGS7* (regulator of G-protein 7); *FH* (fumarate hydratase); *KMO* (kynurenine 3-monooxygenase); *OPN3* (opsin 3); *WDR64* (WD repeat domain 64); *EXO1* (exonuclease 1); *MAP1LC3C (*microtubule-associated protein 1 light chain 3 gamma); *PLD5* (phospholipase D family, member 5).

General linear regression modeling resulted in 11 out of 1,902 SNPs showing promising associations with BMI (10^−5^ < p ≤ 10^−3^) under different genetic models, study groups or in meta-analysis. The strongest associations were observed under a recessive genetic model in the AA group (**Table 1)**; they peaked toward the 3’ end of *RGS7* (**Fig 1**) at intronic rs261802 (p = 1.0 x 10^−4^).

**Table 1:** Association of body mass index with SNP variants in the *RGS7-PLD5* genomic interval in NIEHS-UFS.

	MAF			European Americans (n = 391)			African Americans (n = 525)			meta-analysis (n = 916)	
SNP	EA	AA	gene	Β	SE	p	β	SE	p	p^a^	p-het
**rs261802**	**0.00**	**0.07**	***RGS7***				**0.467**	**0.119**	**1.0E-04**	**9.0E-05**	**1.0E+00**
rs261850	0.00	0.05	*RGS7*	0.119	0.202	5.6E-01	0.527	0.171	2.1E-03	9.8E-02	1.2E-01
rs261862	0.04	0.08	*RGS7*	0.149	0.200	4.6E-01	0.462	0.138	8.8E-04	2.7E-02	2.0E-01
rs9787056	0.24	0.37	*RGS7*	0.062	0.048	2.0E-01	0.085	0.030	4.8E-03	2.1E-03	6.8E-01
rs7543001	0.43	0.37	*RGS7*	0.088	0.027	1.1E-03	0.036	0.031	2.4E-01	1.4E-02	2.0E-01
rs6429264	0.08	0.18	RGS7*	0.311	0.100	2.0E-03	0.075	0.054	1.6E-01	1.3E-01	3.7E-02
rs72632896	0.02	0.08	WDR64*	-0.060	0.072	4.0E-01	-0.498	0.171	3.6E-03	2.5E-01	1.8E-02
rs9428888	0.09	0.28	*WDR64*	0.357	0.114	1.9E-03	0.067	0.036	6.0E-02	1.8E-01	1.5E-02
rs12118937	0.20	0.04	EXO1*	0.182	0.060	2.8E-03				2.6E-03	1.0E+00
rs4658619	0.43	0.40	*PLD5*	-0.074	0.025	2.8E-03	-0.051	0.029	7.6E-02	5.8E-04	5.5E-01
rs12127920	0.47	0.45	*PLD5*	-0.061	0.024	1.1E-02	-0.067	0.026	1.1E-02	3.2E-04	8.6E-01

Footnote to Table 1. List of single nucleotide polymorphisms (SNPs) showing the strongest associations with the body mass index (BMI) under a recessive genetic model. (*) Asterisks indicate SNP locations within the 2-Kb upstream gene sequences; all other SNPs are located in introns. (MAF) minor allele frequency; (β) regression coefficient; (SE) standard error; (p) two-sided p-value; (p^a^) one-sided p-value; (p-het) p-value for the test of heterogeneity. (EA) European Americans and (AA) African Americans. *RGS7* (regulator of G-protein signaling 7); *WDR64* (WD repeat domain 64)*; PLD5* (Phospholipase D family, member 5). (blanks) not estimated. (Boldface) most significantly associated variant.

The minor allele frequency (MAF) of the associated rs261802 variant in AA (MAF = 0.067) and EA (MAF = 0.001) is consistent with that of the reference AA and EA populations of the 1,000 genomes project. The associations in EA also peaked in *RGS7* but at the opposite gene end overlapping about 50 Kb of the first intron at rs7543001 (p = 1.1 x 10^−3^) and the upstream 2 Kb gene sequence at rs6429264 (p = 2.0 x 10^−3^). Moderate associations were also observed in *WDR64* (WD repeat domain 64) at intronic rs9428888 (p = 1.9 x 10^−3^) in EA and upstream 2-Kb rs72632896 (p = 3.6 x 10^−3^) in AA.

SNP associations of similar strengths were also observed in EA in *EXO1* (Exonuclease I) 2-Kb upstream variant rs12118937 (p = 2.8 x 10^−3^) and in *PLD5* (Phospholipase D family, member 5) intronic variant rs4658619 (p = 2.8 x 10^−3^). Analysis of pairwise linkage equilibrium in EA showed weak correlation (D’ = 0.11) between the associated alleles at rs6429264 and rs12118937, suggesting potentially independent associations of *RGS7* and *EXO1* variants in the EA group (not shown). Other SNPs passed the cutoff p ≤ 0.01 but were not listed in Table 1 due to either low MAF in both study populations, strong LD with a listed SNP in Table 1 or SNPs with opposite effects in EA and AA (**S1 Table**).

Compared to GLM analysis, stronger associations were detected at the 3’ends of *RGS7* and *PLD5* in meta-analysis, namely at intronic rs9787056 (p = 2.1 x 10^−3^) and rs12127920 (p = 3.2 x 10^−4^) variants, respectively, whereas losses of association were observed at the 5’end of RGS7 and 3’end of *WDR64* (**Table 1**).

For those SNP associations that were estimated in either EA or AA but not in both, for instance *RGS7* rs261802 (p = 9.0 x 10^−5^) and *EXO1* rs12118937 (p = 2.6 x 10^−3^), the different p-values obtained in race-stratified analysis and in meta-analysis is due to the type of p-values generated in these models (two-sided vs one-sided p-values, respectively). As can be predicted, several SNP associations showing opposing effects in EA and AA cancelled out in meta-analyses (**S1 Table**).

Testing under additive (**S2 Fig and S2 Table**) and dominant (**S3 Fig and S3 Table**) genetic models highlighted similar patterns of association, albeit of smaller magnitude and strength, peaking essentially in *RGS7*.

**Fig 2:**
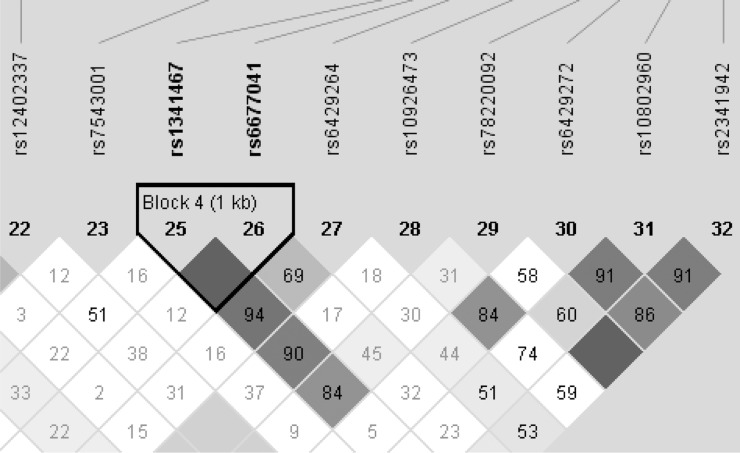
Linkage disequilibrium pattern across the 5’end and upstream sequence of *RGS7* in the 1,000 genome project reference populations for the European ancestry. The plot shows the pairwise linkage disequilibrium (LD) and haploblock structure across a 157 Kb-long interval overlapping the first translated exon of *RGS7* (regulator of G protein signaling 7) on human chromosome 1q43. The haploblock structure was obtained using D’ measures of pairwise LD determined for single nucleotide polymorphism (SNP) genotypes available in reference populations of the 1,000 genomes project for the European ancestry (super European population). The genetic analysis program Haploview was used to estimate D’ and to visualize the structure of haploblocks in the region associated with BMI and body fat in populations of European descent. Note that because the LD pattern may be distorted in the presence of founder effect, the Finish reference population (FIN) was not included in the superpopulation sample used for the construction of the LD plot. Haploblock 4 is located in the 2-Kb upstream sequence of *RGS7* and includes candidate body fat SNP rs1341467 in the Quebec Family Study, which is in strong LD (D’ = 0.94) with the candidate BMI SNP rs6429264 in NIEHS-UFS. Note also that the last four SNPs map to the 20 Kb upstream sequence of *RGS7*; these SNPs are candidates for uterine fibroids that were added to the LD plot to check for potential correlations with the candidate obesity SNPs.

Furthermore, we examined the LD pattern across *RGS7* in the reference European populations of the 1000 genomes project to compare our association results with those reported earlier in QFS [7] because the *RGS7* rs1341467 SNP at which the association with body fat peaked in QFS failed genotyping in the present study. We show that our strongest BMI candidate rs6429264 in EA occurs in strong LD with rs1341467 (D’ = 0.94) in the reference European populations in the 1,000 genomes project (**Fig 2**and **S4 Fig**).

## Discussion

In the present work in NIEHS-UFS, we used BMI as a surrogate measure of obesity to finely map a putative QTL for body fat reported earlier in QFS [7]. We have screened 2.3 Mb of chromosome 1q43 DNA at a high SNP density (about 1 SNP every 1 Kb) in 916 individuals clustered in 2 population groups and showed that the association with BMI peaked at two positions in the 586-Kb long *RGS7* gene. The most significant association mapped at the intronic variant rs261802 near the 3’ end of *RGS7* in African Americans; however, this association did not remain significant at the 5% threshold after correction for multiple testing. Moderate associations were detected in the first intron of *RGS7* and in the 2 Kb upstream sequence of this gene in the EA group. Interestingly, the *RGS7* upstream variant rs6429264 maps within 4.7 Kb distance from rs1341467, the strongest candidate SNP for total body fat in QFS that failed typing in NIEHS-UFS.

Close examination of the LD pattern in the reference populations of the 1000 genomes showed that rs6429264 and rs1341467 occur in strong LD (D’ = 0.94) in Europeans and exhibit moderate LD in Africans (D’ = 0.66). Thus, the LD pattern in the AA and EA populations and our association findings for these study populations relative to QFS, which sampled a population of European descent, are consistent observations.

The observed difference in the association pattern across *RGS7* between AA and EA may reflect true genetic heterogeneity or heterogeneous LD patterns in these populations. Obviously, the low MAF and/or monomorphism of the SNPs around rs261802 in EA precluded our assessment of the association near the 3’ end of *RGS7* in this population.

To our knowledge, this is the first report of replicated associations of *RGS7* with obesity, albeit with BMI and not with total adiposity as reported in the discovery study [7]. *RGS7* showed a moderate association with BMI in QFS and significant association with total adiposity. Although BMI and adiposity are highly correlated, further evaluations of the association of our candidate SNPs with the body fat phenotype may reveal stronger associations and effect size (2.9% for rs2611802 in the present study) that could substantiate the present observations. Further evaluations are indeed important given the limitations of the present study, which are the lack of additional replication samples and measurements of total adiposity; moderate sample size and non-generalizability of the results to men and younger ages.

Importantly, while the present study refined the association to a 5-Kb region overlapping the 5’ regulatory region of *RGS7* in the EA group, none of the tested SNPs remained significant at the corrected threshold. Thus, future replication analyses are required to substantiate our observations. For such a common trait, we were intrigued by the stronger associations obtained under a recessive genetic model and not under the additive or dominant models. Close examination of the data showed that the few homozygotes for the minor allele at rs261802 have BMI values in the range defined as morbid obesity (36 ≤ BMI ≤ 51).

Of note, although variants on chromosome 1q43 have been associated with body composition and obesity-associated metabolic traits, to our knowledge specific associations with *RGS7* variants have not been reported to date. Several factors might have improved the power of our study to detect associations with *RGS7*. First, we used a dense set of multi-population tag SNPs selected in a two-stage next-generation sequencing and genotyping study [13, 16]; thus most of the genetic variation in the studied region in these populations is predicted to be captured by our SNP selection. Second, because all the studied individuals are randomly-selected premenopausal women in the 35–51 years range of age; the higher correlation between BMI and body fat among women compared to men [21] likely improved the power to detect the association with the correlated body fat QTL reported in QFS. Furthermore, the sampling of random adult subjects in NIEHS-UFS likely refined the homogeneity of the BMI phenotype (e.g. no interference with the hormonal changes associated with adolescence and post-menopause). Third, we tested the association in a multi-ethnic study and identified different regions of association within the same gene; this diminishes the likelihood of biased findings due to genotyping artifacts (e.g. caused by population-specific copy number variation etc…). Fourth, the association with the BMI outcome was adjusted for the effects of physical activity allowing a more accurate assessment of genetic effects [22].

Several genes on chromosome 1q43 have been associated with obesity- or body composition-related traits through either GWA studies or experimental studies in mice. For instance, *SDCCAG8* (serologically defined colon cancer antigen 8), a gene located about 700 Kb upstream of *PLD5*, is robustly associated with body fat in Europeans [11] and *FMN2* (formin 2), which maps downstream of *RGS7*, is associated with mineral bone density [23]. Experimental studies have shown that mice lacking *CHRM3* (muscarinic acetylcholine receptor 3), the closest gene upstream of *FMN2*, are hypophagic and lean [24], and heterozygous knockout mice for *BECN2* (beclin 2), a 2 Kb-long intronless gene located between *EXO1* and *MAP1LC3C* (microtubule-associated protein 1 light chain 3 gamma), have elevated food intake, obesity and insulin resistance [25]. We observed no associations (p < 0.01) with individual *BECN2* SNPs in the present study.

Very little is known about the function of *PLD5*. Members of the PLD family regulate downstream molecules by generating phosphatidic acid (PA), a biologically active product widely considered to be the intracellular lipid mediator of many of the biological functions attributed to PLD. The best studied members of this family, *PLD1* and *PLD2*, have been implicated in several pathophysiological processes; however, enzymatic activities have not been found for the products encoded by known paralogs including *PLD5* [26]. To date *PLD5* has not been associated with anthropometric measures of obesity but evidence for suggestive association of *PLD5* with a marker of obesity (serum estradiol) has been reported [9].

*RGS7* encodes a member of the R7 family of regulators of G-protein signaling (RGS) proteins ([27] for a review), which are primarily known as GTPase-activating proteins (GAPs) for heterotrimeric G proteins. The ability to respond promptly to a rapidly changing milieu necessitates timely inactivation of G proteins, a process controlled by RGS proteins. Four proteins in the R7 group (RGS6, RGS7, RGS9, and RGS11) share a common trimeric structure composed of a catalytic RGS domain, a GGL domain that recruits Gβ5, an outlying member of the G protein β subunit family, and a DEP/DHEX domain that mediates interactions with the membrane anchor proteins R7BP and R9AP. *RGS7* is abundantly expressed in the brain, particularly in the hypothalamus and might be involved in the regulation of hypothalamic-pituitary-adrenal axis responses to diverse stresses and stimuli.

Despite the above reports suggesting the presence of putative obesity gene loci other than *RGS7* in 1q43, studies using different approaches led supports for *RGS7* as a candidate body fat QTL in this chromosomal region. First, in addition to the anchor R7BP and R9AP interaction partners, RGS7 forms also complexes with non-G protein molecules implicated in adiposity; for instance with 14-3-3 [28], a coordinator of adipogenesis in visceral fat [29], and with muscarinic 3 receptor (M3R) [24, 30], which is encoded by the *RGS7*-linked *CHRM3* on chromosome 1q43. Second, *RGS9*, another member of the R7 family highly expressed in the brain has been shown to associate with BMI in humans and with body weight reduction in virally transduced overexpression of *RGS9-2* in rats [31]; though opposite effects were reported in similar experiments with the related *RGS7* or *RGS11* members. The opposing actions of *RGS7* and *RGS11* on body weight are thought to result from competitions between the R7 RGS proteins for their obligate partners Gβ5 subunit [31]. Gβ5 protects R7 family members from proteolysis and expression of the latter, including RGS9, is eliminated or heavily reduced in the absence of Gß5 [32]. Because mice with a targeted deletion of one copy of Gß5 are heavier and have increased adiposity compared to wild-type mice [33], Waugh and colleagues suggested that the increased adiposity and weight phenotype of heterozygous Gß5 knockout mice is produced as a result of a Gß5 knockdown-mediated reduction in brain RGS9-2 protein levels [31]. Third, *RGS7* is part of a QTL hotspot in the syntenic region of mouse chromosome 1 implicated in obesity and behavioral phenotypes [34]; it is quite possible that a similar body composition QTL hotspot exist on human chromosome 1q43.

The present study was motivated by previous observations suggesting a role for this genomic region in the development of obesity and uterine leiomyoma (fibroid tumors) [7, 13, 16, 35, 36]. Furthermore, because obesity is associated with fibroids, albeit in a complex relationship (inverse J-shaped association peaking in the overweight category) [14, 37], models for the observed co-localization of reproduction- and body composition-related traits and diseases to chromosome 1q43 have been proposed [38]; these were i) presence of a pleiotropic adiposity gene affecting the risk for fibroids through changes to steroid bioavailability as a result of decreasing serum level of sex hormone-binding globulin, a regulation of which has been linked to a QTL locus in the *RGS7* region [39], ii) LD between variants in two distinct genes with independent influences on obesity and fibroids, and iii) coordinated expression of major reproductive and metabolic genes influencing thrifty correlated phenotypes. Our recent fine mapping of the fibroid locus on 1q43 close to a long non-coding RNA (lncRNA) locus located between *RGS7* and *FH* (fumarate hydratase) [16], and the report of co-regulated expressions of the same lncRNA and *RGS7* in fibroids [40] are potentially consistent with the hypothesis of a pleiotropic *RGS7*.

In conclusion, we reported for the first time data suggesting that *RGS7*, a gene abundantly expressed in the hypothalamus, might be a body fat QTL on human chromosome 1q43. Future genetic and functional studies are required to validate our results.

## Supporting Information

S1 Fig Principal Component Analysis showing clustering of the NIEHS-UFS study populations relative to the reference populations of the International HapMap III project. The plot shows the results of Discriminant Analysis of Principal Components (DAPC) 1 and 2 to define clusters of genetically related individuals. A total set of 2,682 SNPs that were common to our data and to the majority of the HapMap III reference populations was used to re-assess the population membership of each of 1,003 individual of NIEHS-UFS (large blue diamonds) with genotyping call rates > 95%. The three ethnic groups (African Americans, European Americans and “other”) to which the sampled individuals self-identified clustered with the African (YRI, LWK and MKK) and African American (ASW) populations, the European populations and the more scattered group composed of Asians, Hispanics and individuals of mixed origin, respectively.
(DOCX)

S2 Fig Fine genetic association map of body mass index on chromosome 1q43 in the NIEHS uterine fibroid study (additive model). The plot shows the strength of association (expressed as minus log_10_ of p-value) of the body mass index (BMI) with a dense set (n = 1,902) of single nucleotide polymorphisms (SNPs) in a sample of 391 European American (EA) and 525 African American (AA) women participants to NIEHS-UFS (National Institute of Environmental Health Science-Uterine Fibroid Study). P-values were obtained from general linear models adjusted for covariates (age, physical activity and uterine fibroid affection status). The results were obtained under the assumption of an additive genetic model. Meta-analysis of EA and AA samples was conducted in the R package Metafor using random-effect variance and the Dersimonian-Laird estimator. *RGS7* (regulator of G-protein 7); *FH* (fumarate hydratase); *KMO* (kynurenine 3-monooxygenase); *OPN3* (opsin 3); *WDR64* (WD repeat domain 64); *EXO1* (exonuclease 1); *MAP1LC3C (*microtubule-associated protein 1 light chain 3 gamma); *PLD5* (phospholipase D family, member 5).
(DOCX)

S3 Fig Fine genetic association map of body mass index on chromosome 1q43 in the NIEHS uterine fibroid study (dominant model). The plot shows the strength of association (expressed as minus log_10_ of p-value) of the body mass index (BMI) with a dense set (n = 1,902) of single nucleotide polymorphisms (SNPs) in a sample of 391 European American (EA) and 525 African American (AA) women participants to NIEHS-UFS (National Institute of Environmental Health Science-Uterine Fibroid Study). P-values were obtained from general linear models adjusted for covariates (age, physical activity and uterine fibroid affection status). The results were obtained under the assumption of a dominant genetic model. Meta-analysis of EA and AA samples was conducted in the R package Metafor using random-effect variance and the Dersimonian-Laird estimator. *RGS7* (regulator of G-protein 7); *FH* (fumarate hydratase); *KMO* (kynurenine 3-monooxygenase); *OPN3* (opsin 3); *WDR64* (WD repeat domain 64); *EXO1* (exonuclease 1); *MAP1LC3C (*microtubule-associated protein 1 light chain 3 gamma); *PLD5* (phospholipase D family, member 5).
(DOCX)

S4 Fig Linkage disequilibrium pattern across RGS7 in the 1,000 genome project reference populations of European descent. The plot shows the pairwise linkage disequilibrium (LD) and haploblock structure across the 586 Kb-long *RGS7* (regulator of G protein signaling 7) on human chromosome 1q43. The haploblock structure was obtained using D’ measures of pairwise LD determined for single nucleotide polymorphism (SNP) genotypes available in reference populations of the 1,000 genomes project for the European ancestry (super European population). The genetic analysis program Haploview was used to estimate D’ and to visualize the structure of haploblocks in the region associated with BMI and body fat in populations of European descent. Note that because the LD pattern may be distorted in the presence of founder effect, the Finish reference population (FIN) was not included in the superpopulation sample used for the construction of the LD plot. Haploblock 4 is located in the 2-Kb upstream sequence of *RGS7* and includes candidate body fat SNP rs1341467 in the Quebec Family Study, which is in strong LD (D’ = 0.94) with the candidate BMI SNP rs6429264 in NIEHS-UFS. Note also that the last four SNPs map to the 20 Kb upstream sequence of *RGS7*; these SNPs are candidates for uterine fibroids; they were added to the LD plot to check for potential correlations with the candidate obesity SNPs.
(PNG)

S1 Table Association of body mass index with SNP variants in the *RGS7-PLD5* interval in NIEHS-UFS (recessive genetic model). Only single nucleotide polymorphisms (SNPs) showing association at p ≤ 0.01 in any model, study group or in meta-analysis are listed; (MAF) minor allele frequency; (beta) regression coefficient; (SE) standard error; (p) two-sided p-value in race-stratified analysis and one-sided p-value in meta-analysis; (p-het) p-value for the test of heterogeneity. (blank) not estimated. EA (European Americans; n = 391); AA (African American; n = 525). *RGS7* (regulator of G-protein signaling 7); *WDR64* (WD repeat domain 64); *EXO1* (exonuclease 1); *PLD5* (Phospholipase D family, member 5).
(XLSX)

S2 Table Association of body mass index with SNP variants in the *RGS7-PLD5* interval in NIEHS-UFS (additive genetic model). Only single nucleotide polymorphisms (SNPs) showing association at p ≤ 0.01 in any model, study group or in meta-analysis are listed; (MAF) minor allele frequency; (beta) regression coefficient; (SE) standard error; (p) two-sided p-value in race-stratified analysis and one-sided p-value in meta-analysis; (p-het) p-value for the test of heterogeneity. (blank) not estimated. EA (European Americans; n = 391); AA (African American; n = 525). *RGS7* (regulator of G-protein signaling 7); *FH* (fumarate hydratase); *WDR64* (WD repeat domain 64); *EXO1* (exonuclease 1); *PLD5* (Phospholipase D family, member 5).
(XLSX)

S3 Table Association of body mass index with SNP variants in the *RGS7-PLD5* interval in NIEHS-UFS (dominant genetic model). Only single nucleotide polymorphisms (SNPs) showing association at p ≤ 0.01 in any model, study group or in meta-analysis are listed; (MAF) minor allele frequency; (beta) regression coefficient; (SE) standard error; (p) two-sided p-value in race-stratified analysis and one-sided p-value in meta-analysis; (p-het) p-value for the test of heterogeneity. (blank) not estimated. EA (European Americans; n = 391); AA (African American; n = 525). *RGS7* (regulator of G-protein signaling 7); *FH* (fumarate hydratase); *WDR64* (WD repeat domain 64); *EXO1* (exonuclease 1); *PLD5* (Phospholipase D family, member 5).
(XLSX)
