# Supplementary figures and images for: Fine Mapping of the Body Fat QTL on Human Chromosome 1q43
Source: PLoS One. 2016 Apr 25;11(4):e0153794. doi: 10.1371/journal.pone.0153794 (PMC4844098; doi:10.1371/journal.pone.0153794)

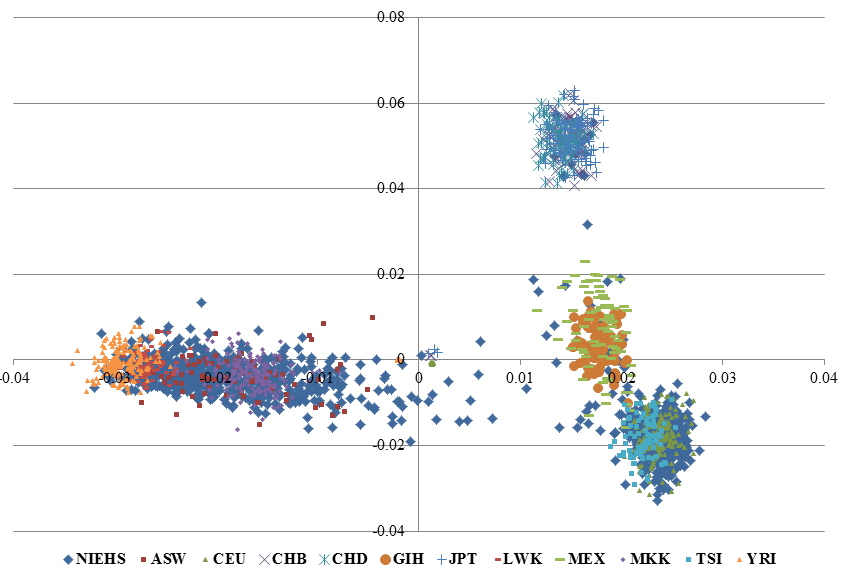

Supplement: S1 Fig — The plot shows the results of Discriminant Analysis of Principal Components (DAPC) 1 and 2 to define clusters of genetically related individuals. A total set of 2,682 SNPs that were common to our data and to the majority of the HapMap III reference populations was used to re-assess the population membership of each of 1,003 individual of NIEHS-UFS (large blue diamonds) with genotyping call rates > 95%. The three ethnic groups (African Americans, European Americans and “other”) to which the sampled individuals self-identified clustered with the African (YRI, LWK and MKK) and African American (ASW) populations, the European populations and the more scattered group composed of Asians, Hispanics and individuals of mixed origin, respectively. (DOCX) [file pone.0153794.s001.docx]

**S2 Figure**


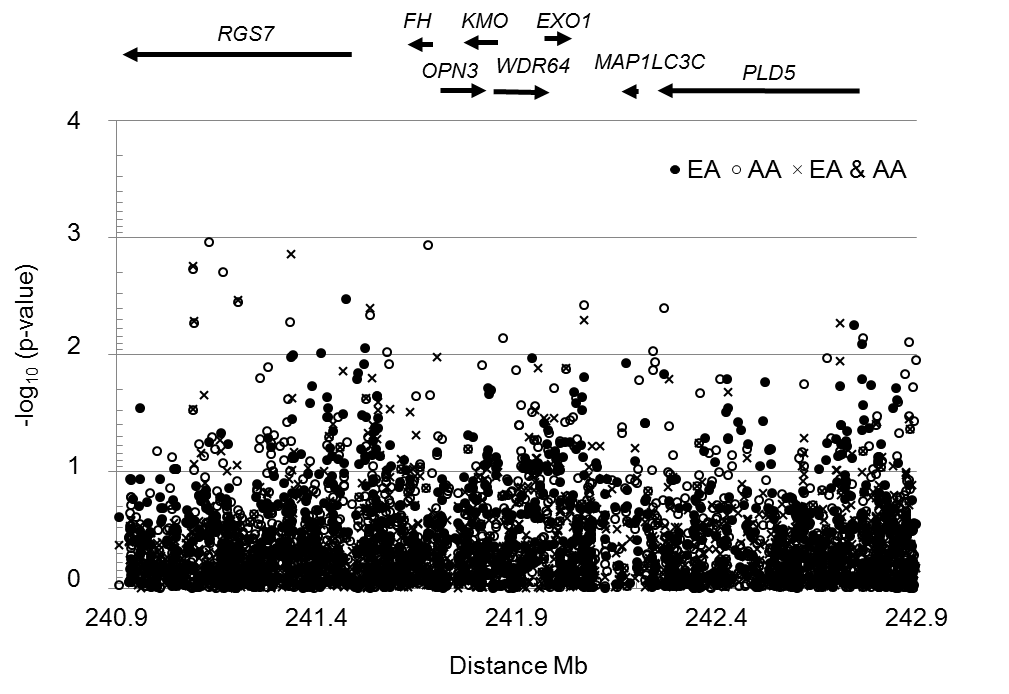

Supplement: S2 Fig — The plot shows the strength of association (expressed as minus log10 of p-value) of the body mass index (BMI) with a dense set (n = 1,902) of single nucleotide polymorphisms (SNPs) in a sample of 391 European American (EA) and 525 African American (AA) women participants to NIEHS-UFS (National Institute of Environmental Health Science-Uterine Fibroid Study). P-values were obtained from general linear models adjusted for covariates (age, physical activity and uterine fibroid affection status). The results were obtained under the assumption of an additive genetic model. Meta-analysis of EA and AA samples was conducted in the R package Metafor using random-effect variance and the Dersimonian-Laird estimator. RGS7 (regulator of G-protein 7); FH (fumarate hydratase); KMO (kynurenine 3-monooxygenase); OPN3 (opsin 3); WDR64 (WD repeat domain 64); EXO1 (exonuclease 1); MAP1LC3C (microtubule-associated protein 1 light chain 3 gamma); PLD5 (phospholipase D family, member 5). (DOCX) [file pone.0153794.s002.docx]

**S3 Figure**


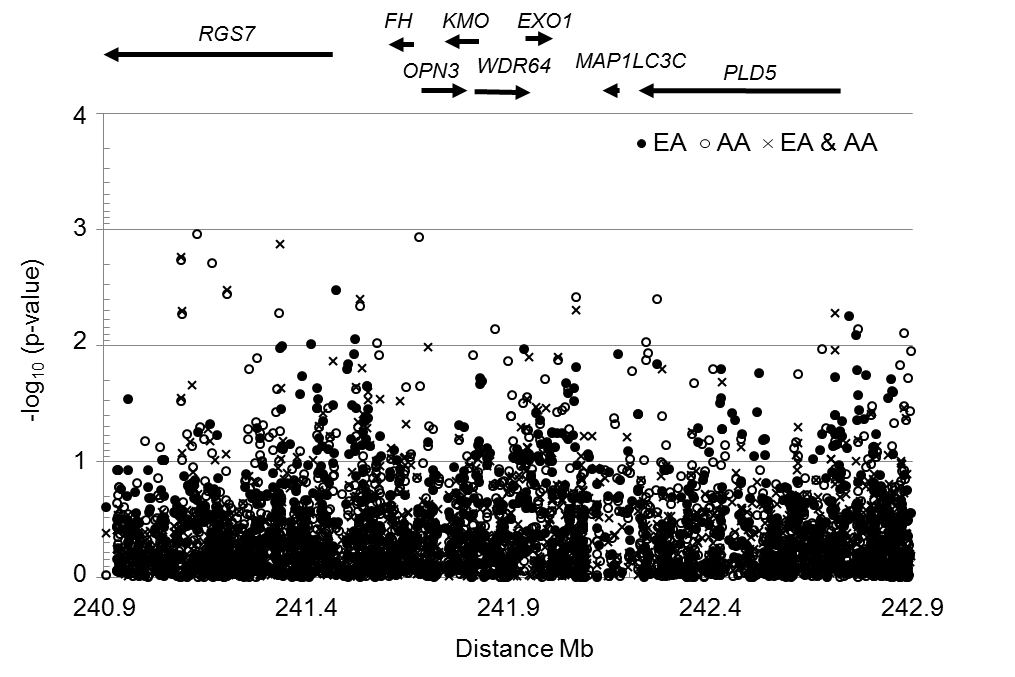

Supplement: S3 Fig — The plot shows the strength of association (expressed as minus log10 of p-value) of the body mass index (BMI) with a dense set (n = 1,902) of single nucleotide polymorphisms (SNPs) in a sample of 391 European American (EA) and 525 African American (AA) women participants to NIEHS-UFS (National Institute of Environmental Health Science-Uterine Fibroid Study). P-values were obtained from general linear models adjusted for covariates (age, physical activity and uterine fibroid affection status). The results were obtained under the assumption of a dominant genetic model. Meta-analysis of EA and AA samples was conducted in the R package Metafor using random-effect variance and the Dersimonian-Laird estimator. RGS7 (regulator of G-protein 7); FH (fumarate hydratase); KMO (kynurenine 3-monooxygenase); OPN3 (opsin 3); WDR64 (WD repeat domain 64); EXO1 (exonuclease 1); MAP1LC3C (microtubule-associated protein 1 light chain 3 gamma); PLD5 (phospholipase D family, member 5). (DOCX) [file pone.0153794.s003.docx]

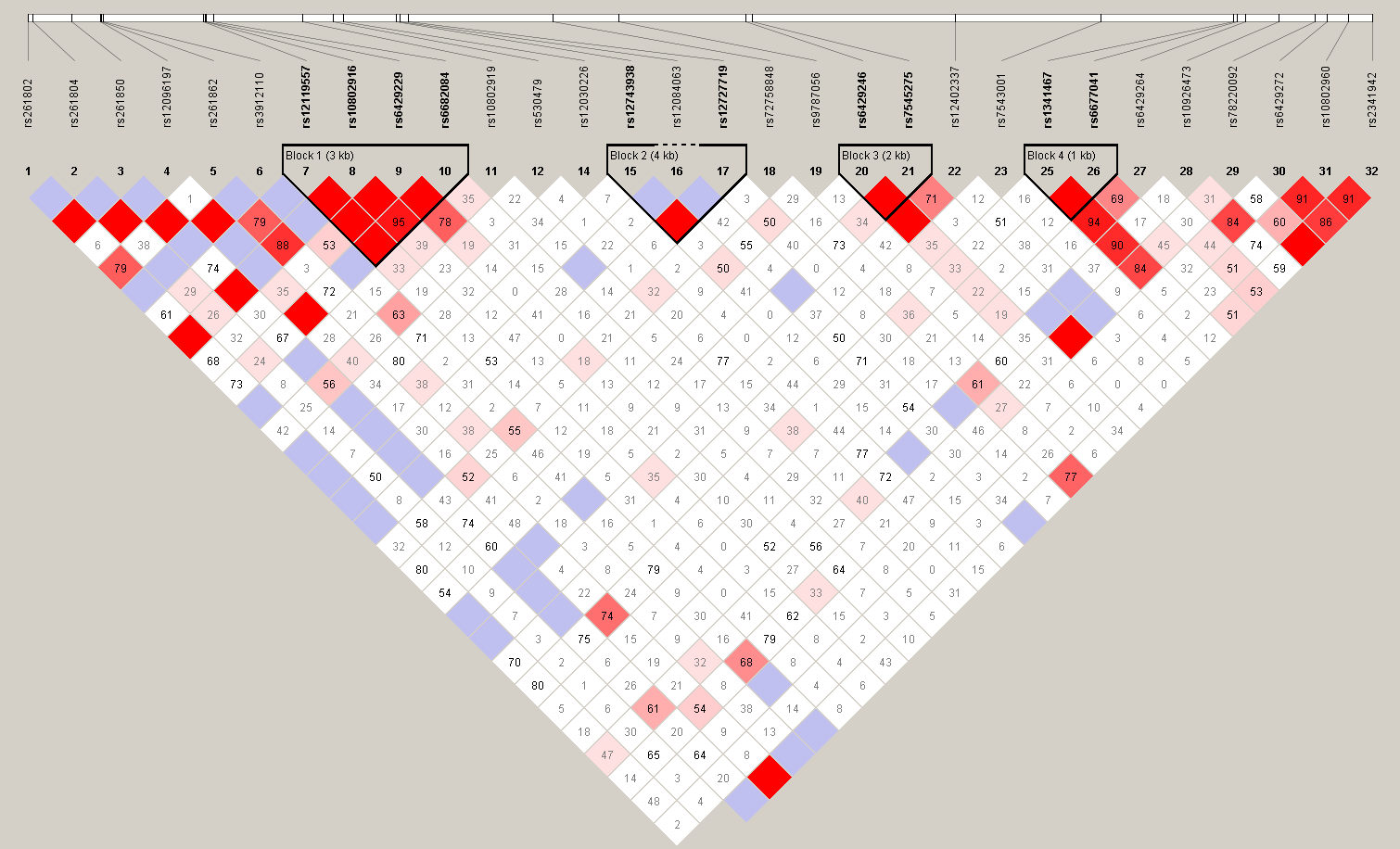

Supplement: S4 Fig — The plot shows the pairwise linkage disequilibrium (LD) and haploblock structure across the 586 Kb-long RGS7 (regulator of G protein signaling 7) on human chromosome 1q43. The haploblock structure was obtained using D’ measures of pairwise LD determined for single nucleotide polymorphism (SNP) genotypes available in reference populations of the 1,000 genomes project for the European ancestry (super European population). The genetic analysis program Haploview was used to estimate D’ and to visualize the structure of haploblocks in the region associated with BMI and body fat in populations of European descent. Note that because the LD pattern may be distorted in the presence of founder effect, the Finish reference population (FIN) was not included in the superpopulation sample used for the construction of the LD plot. Haploblock 4 is located in the 2-Kb upstream sequence of RGS7 and includes candidate body fat SNP rs1341467 in the Quebec Family Study, which is in strong LD (D’ = 0.94) with the candidate BMI SNP rs6429264 in NIEHS-UFS. Note also that the last four SNPs map to the 20 Kb upstream sequence of RGS7; these SNPs are candidates for uterine fibroids; they were added to the LD plot to check for potential correlations with the candidate obesity SNPs. (PNG) [file pone.0153794.s004.png]
